# An Interesting Case of Fatal Myasthenic Crisis Probably Induced by the COVID-19 Vaccine

**DOI:** 10.7759/cureus.23251

**Published:** 2022-03-17

**Authors:** Khushboo J Sonigra, Krishan Sarna, Vinesh P Vaghela, Symon Guthua

**Affiliations:** 1 Department of Oral and Maxillofacial Surgery, Oral Pathology and Oral Medicine, University of Nairobi, Nairobi, KEN; 2 Department of Internal Medicine, Coast General Teaching and Referral Hospital, Mombasa, KEN

**Keywords:** astrazeneca vaccine, vaccination, covid-19, myocardial infarction, myasthenic crisis, myasthenia gravis

## Abstract

A myasthenic crisis is a severe, life-threatening exacerbation of myasthenia gravis that causes a rapid onset of muscle weakness and fatigue that may result in tetraparesis, dyspnea, respiratory insufficiency, aspiration, and death. Bulbar muscle functions are markedly affected resulting in depressed cough reflex, swallowing, and speech. Thus, mechanical ventilation, supportive feeding, and critical care are essential for the survival of patients in a myasthenic crisis. Numerous precipitating factors of this condition are well known and include infections, various medications, pregnancy, and childbirth. Patients with myasthenia gravis are at a considerably higher risk of developing a debilitating coronavirus disease 2019 (COVID-19) infection due to the associated immunosuppression resulting from long-term corticosteroid use, which makes vaccination of such individuals necessary. However, the relationship between an exacerbation of myasthenia gravis and the COVID-19 vaccination is currently unknown. In this paper, we report the case of a 55-year-old male patient who developed a myasthenic crisis after receiving the first dose of the ChAdOx1-S (recombinant) vaccine (AstraZeneca batch number 210157; AstraZeneca plc, Cambridge, United Kingdom). Despite the administration of aggressive and intensive treatment over a period of 29-day hospitalization, the myasthenic crisis could not be reversed and the patient ultimately deteriorated and succumbed from multiple myocardial infarction events and organ failures. While it is still uncommon, evidence associating the effects of the vaccine to the development of a crisis is mounting; therefore, it is crucial for clinicians to promptly identify clinical features that suggest an exacerbation of myasthenia gravis in order to intervene at the earliest possible stage for a more favorable outcome. The myasthenia gravis patient should be informed about the possible association between COVID-19 vaccination and the development of a myasthenic crisis.

## Introduction

Myasthenia gravis (MG) is a rare autoimmune neurological disorder characterized by defective transmission at the neuromuscular junction (NMJ) resulting in fatigable muscle weakness of isolated or multiple muscle groups that becomes worse with exertion but improves with rest [1,2]. It has an incidence of 0.04-5.0/100,000 per year and is known to affect women between the ages of 20 and 30, and males aged 50 and older [3]. The pathogenesis of the disease stems from the generation of autoantibodies against the acetylcholine receptors (AChRs) at the postsynaptic membrane of skeletal muscles. In other less frequent phenotypic variants of MG, antibodies against the muscle-specific kinase (MuSK) receptor, low-density lipoprotein (LDL) receptor-4, or agrin have also been identified. The detection of these various types of antibodies forms the basis of categorizing the various types of MG that have been described in the past [4]. Clinically, initial ocular symptoms such as diplopia and ptosis are most common; however, 80% may go on to develop generalized MG characterized by bulbar muscle involvement resulting in flaccid dysarthria, dysphagia, and facial and jaw weakness [5]. Additionally, axial weakness manifested as a ‘head-drop’ and proximal limb weakness may also be present. Certain precipitating factors such as systemic infections, pregnancy, antibiotics, metabolic disorders, and sedatives may result in an acute exacerbation of MG, which is manifested by progressive respiratory and bulbar muscle weakness leading to acute decompensation with aspiration and respiratory failure [6,7]. This life-threatening complication of MG is known as a myasthenic crisis. Prompt detection of signs and symptoms coupled with intervention consisting partly of intubation, mechanical ventilation, and supportive feeding is essential in the survival of such patients.

During the current coronavirus disease 2019 (COVID-19) pandemic, patients with pre-existing neuromuscular and autoimmune diseases are seen to be at a significantly greater risk of not only contracting the virus but developing more severe, debilitating infections that are associated with a poorer prognosis and higher mortality rate [8]. It is hypothesized that aggravation of such conditions may be attributed to the cross-reactivity of antibodies generated in response to viral surface epitopes with the proteins present at the NMJ. Indeed, studies have shown that patients with pre-existing MG who contract the COVID-19 infection, may present with respiratory distress syndrome, functional disability even in absence of physical activity, cerebrovascular and cardiovascular diseases, and immune-mediated neuropathy [8]. Vaccination against COVID-19 reduces the severity of the infection and is associated with a better outcome for such patients [9]. However, there is a dearth of information regarding the relationship between the COVID-19 vaccine and the exacerbation of MG in patients who were previously stable and compliant on medication. Here, we describe a case of a 55-year-old male who developed a fatal myasthenic crisis suspected to be induced by the COVID-19 vaccine.

## Case presentation

A 55-year-old Kenyan male, diagnosed in the past with generalized MG, diabetes, and hypertension presented to the emergency room (ER) with breathlessness, chest pain, profuse sweating, and cold clammy extremities. Regarding the past medical history, the patient was first diagnosed with MG 18 years ago and was maintained on prednisone 15 mg tablet every alternate day, pyridostigmine 60 mg once daily and azathioprine 150 mg once daily. Management of diabetes was achieved using mixtard human insulin 15 units in the morning and 10 units in the evening and metformin 1g twice daily. Losartan 50 mg once daily was used to control hypertension. The patient was compliant on all medications. Two weeks after taking the first dose of the ChAdOx1-S (recombinant) vaccine (AstraZeneca batch number 210157; AstraZeneca plc, Cambridge, United Kingdom), the patient reported dysphagia, dysarthria, and breathlessness for a further period of three weeks during which the condition of the patient deteriorated as the symptoms worsened. The frequency of pyridostigmine was increased to every two hourly (12 times a day) upon instruction by the neurologist. However, this did not reverse the condition.

Upon presentation to the ER, vitals were significant for hypoxia at 69% (room air), tachycardia at 145 beats per minute, and tachypnea at 40 breaths per minute with the unfavorable outcome of cardiac arrest but was successfully resuscitated. The ECG revealed sinus tachycardia and non-ST-elevated myocardial infarction (N-STEMI) while subsequent echocardiogram showed a hypokinetic anterior wall, interventricular septum, and apex. The chest x-ray was negative for any infiltrates, effusions, or consolidations. A full hemogram revealed decreased red blood cell counts and low levels of both hemoglobin and hematocrit while the platelet count was found to be elevated (Table 1).

**Table 1:** Results of the full hemogram showing decreased levels of red blood cells, hemoglobin, and hematocrit. Platelet count and plateletcrit were significantly elevated.

PARAMETER	RESULT	UNITS	RANGE
White blood cells	8.41	×10^9^/L	4 - 11
Neutrophils	65.9	%	45 - 75
Lymphocytes	26.4	%	20 - 45
Monocytes	5.7	%	2 - 10
Eosinophils	1.6	%	1 - 6
Basophils	0.4	%	0 - 1
Red blood cells	3.69	× 10^12^/L	4.5 - 6.5
Hemoglobin	10.9	g/dL	13.5 - 18
Hematocrit	34.4	%	40 - 54
Mean Cell Volume	93.2	fl	76 - 96
Mean Corpuscular Hemoglobin	29.4	pg	27 - 34
Mean Corpuscular Hemoglobin Concentration	32.6	g/dL	32 - 36
Platelet count	534	× 10^9^/L	150 - 400
Plateletcrit	0.435	g/dL	0.108 - 0.282

 Urea, electrolytes, creatinine, and C-reactive protein (CRP) were all within normal ranges (Table 2).

**Table 2:** Urea, electrolytes, and creatinine were found to be within normal ranges at presentation to the ER

TEST	RESULT	UNITS	RANGE
Urea	2.5	mmol/l	1.7 - 8.3
Sodium (Na^+^)	138	mmol/l	135 - 148
Potassium (K^+^)	4.6	mmol/l	3.5 - 5
Chloride (Cl^-^)	106	mmol/l	95 - 108
Bicarbonate (HCO^3-^)	23	mmol/l	24 - 33
Creatinine	55	umol/l	50 - 115
C-Reactive Protein (CRP)	8	mg/l	4 - 9
D-dimers	0.21	mg/l	0 - 0.5

The COVID-19 reverse transcriptase-polymerase chain reaction (RT-PCR) test was negative throughout. Due to difficulty in breathing, the patient was immediately intubated and admitted to the intensive care unit (ICU) for observation and treatment of a myasthenic crisis with concomitant myocardial infarction. The patient was started on enoxaparin sodium 60 mg twice daily for eight days.

After 24 hours, the patient was extubated successfully; however, the dysphagia and dysarthria persisted. During this time the patient developed severe diarrhea characterized by a tremendous decline in blood pressure and tidal volumes. A blood-gas analysis revealed slight compensated metabolic acidosis accompanied by an increased level of glucose (Table 3). 

**Table 3:** Results of the blood-gas analysis revealing an acidic blood pH accompanied by low pO2, low oximetry values, and cHCO3- levels. The glucose levels were found to be high. pCO2: partial pressure of carbon dioxide; pO2: partial pressure of oxygen; SpO2: oxygen saturation; cHCO3-: concentration of plasma bicarbonate; ctBil: change in total bilirubin; FO2Hbe: fractional oxyhemoglobin

TEST	RESULT	UNITS	RANGE
pH	7.346		7.35 - 7.45
pCO_2_	4.3	kPa	4.2 - 6.0
PO_2_	9.8	kPa	10.0 - 13.3
Oximetry values			
ctHemoglobin	10.9	g/dL	11.0 – 18.0
Hematocrit c	34.5	%	38.0 – 54.0
SpO_2_	72	%	96.0 – 99.0
FO_2_Hb_e_	80	%	90.0 – 95.0
Metabolite values			
cGlucose	11.0	mmol/L	4.0 – 8.3
cLactate	1.0	mmol/L	0.2-2.2
ctBil	9	umol/L	4 - 21
Acid Base Status			
cHCO^3-^	22.0	mmol/L	24.0 – 28.0

The septic screen was negative. Re-intubation was performed and a dopamine infusion (1 microgram per kilogram per minute) was started in conjunction with intravenous fluids (4L of normal saline 24 hourly) as a response to an ejection fraction of 28%, low blood pressure, and tachycardia, all of which tended towards cardiogenic shock. Five days later, the patient was weaned off the dopamine infusion. Extubation was again attempted but was unsuccessful. The patient was started on prednisone 30 mg tablet once daily and pyridostigmine 60 mg tablet two hourly. Intravenous immunoglobulin (IVIG) infusion 35 g daily for five days was also commenced. Over a span of the next two weeks, the condition remained unchanged. However, after this time period, the patient developed an event of an ST-elevated myocardial infarction (STEMI). This further deteriorated the patient’s condition, which was manifested by severe lethargy and flaccidity. A percutaneous tracheostomy was performed in an attempt to wean off the ventilator. The patient later developed a fever of 38.8°C and a decline in blood pressure and oxygen saturation (80%). A repeat chest x-ray showed opacification bilaterally in the lung base, which was consistent with a pneumonia-like picture. At this stage, a repeat RT-PCR was performed, which was still negative. Intravenous meropenem 1g thrice a day was initiated to treat the infection. A few days later, the patient developed septic shock characterized by a blood pressure of 70/37 mmHg and the blood culture showed, the multi-drug resistant* Acinetobacter baumannii* but sensitive to piperacillin/tazobactam, which was administered every six hours (4.5g). However, the patient sustained three further myocardial infarctions the next day leading to death.

## Discussion

In the COVID-19 pandemic, individuals with pre-existing health conditions such as MG are at a potentially higher risk for developing severe forms of infection requiring hospitalization and intensive care without which the outcome may be unfavorable [8]. This has indeed been observed by numerous studies around the world hence necessitating vaccination of such patients against COVID-19 in an effort to lessen the impact of infection and improve outcomes. Recent literature, however, has painted a picture of the COVID-19 vaccine as being a ‘double-edged sword’ in the case of patients with autoimmune diseases in that although it offers immunity against the disease, it may, in some cases, aggravate the otherwise stable condition [10]. Exacerbation of autoimmune conditions by a vaccine was only thought to be possible theoretically, however, with the description of this case and that by Tagliaferri et al., 2021, further investigation on the subject is warranted [11]. Although a cross-sectional study by Ruan et al., 2021, stated that the majority of MG patients did not present with significant worsening of symptoms after COVID-19 vaccination, two patients in the study did develop exacerbations of the disease, and while the symptoms were mild and short-lived, it is reasonable to question the interaction of the vaccine with MG exacerbation [12].

Striking similarities exist between the present case and that described by Tagliaferri et al. [11] even though the patient in that study received the mRNA-1273 vaccine (Moderna; Moderna, Inc., Cambridge, Massachuchetts) while our patient received the ChAdOx1-S (recombinant) vaccine (AstraZeneca, batch number 210157). Both patients presented with dysphagia within a few weeks of taking the first dose of the vaccine, which seems to be the first sign of disease exacerbation. Following admission, both patients had comparable treatment: pyridostigmine and prednisone; both were intubated, adequate rehydration with IV fluids was provided, and IVIG was given for five days. In their study, the patient was successfully extubated after these interventions; however, sepsis, respiratory failure, and myocardial infarctions further complicated the present case, which we believe may have been induced by the COVID-19 vaccine [11,12].

The plausible explanations for the induction of a myasthenic crisis by the COVID-19 vaccine may be attributed to two possible mechanisms: molecular mimicry, and bystander activation. In the former, antigens produced from the mRNA vaccine are recognized by the body as being similar to host tissue antigens resulting in the formation of antibodies that are cross-reactive to both the COVID-19 virus as well as proteins present at the post-synaptic membrane such as AChRs, MuSK receptor, LDL receptor-4, or agrin [13]. In the bystander hypothesis, a previously existing self-antigen is released after activation of the immune system by the vaccine thus resulting in activation of T-cells. This immune response leads to enhanced targeting and destruction of proteins at the NMJ, resulting in further deterioration of the condition. Given the present case, the bystander hypothesis is more applicable due to the existing diagnosis of MG; however, molecular mimicry may have also been involved in the exacerbation of the disease [13,14].

Our patient suffered multiple episodes of myocardial infarctions, which could have been a sequela of the myasthenic crisis. However, it is prudent to question whether the vaccine could have led to such an event considering that previous studies reveal an increased risk of developing both thrombotic events and cardiac arrhythmias following vaccination [15]. The pathophysiology behind this is a controversy with no definitive mechanism identified as of yet. Some authors report that a cytokine storm occurs resulting in systemic inflammatory response syndrome (SIRS) that causes diffuse activation of pro-coagulant factors after administration of the vaccine while others describe the generation of antibodies that attack platelet activators hence increasing the risk of thrombosis in the body [16,17,18]. Yet another hypothesis for thrombosis is that an MG crisis in itself can cause severe stress on the heart resulting in Takotsubo cardiomyopathy, which leads to heart failure with difficulty weaning off the ventilator despite aggressive therapy [1]. High-dose IVIG in MG crisis patients is also known to induce a hypercoagulable state that is associated with N-STEMI and STEMI [19]. Additionally, pyridostigmine, which the patient was on during the MG crisis, is known to cause vasoconstriction of the left anterior descending coronary artery leading to diffuse spasms and obstructive myocardial infarctions [20]. While it is debatable which of these mechanisms played the greatest role in the outcome of this patient, it is difficult to attribute any other factor apart from the COVID-19 vaccine to the initial exacerbation of MG, which was otherwise stable for the past 18 years.

## Conclusions

Induction of a myasthenic crisis by the COVID-19 vaccine seems to be underreported but is extremely challenging to manage, the prognosis of which is highly variable depending on the confounding systemic health conditions of the patient. Clinicians must provide enhanced monitoring of MG patients after vaccination, and intervene at the earliest possible time when initial symptoms of the disease begin to appear despite being well managed previously. The patient should also be informed that there could be an association between the COVID-19 vaccine and unfavorable outcomes such as MG exacerbation for patients with MG.

## References

[1] Gilhus NE (2016). Myasthenia gravis. N Engl J Med.

[2] Conti-Fine BM, Milani M, Kaminski HJ (2006). Myasthenia gravis: past, present, and future. J Clin Invest.

[3] Nair AG, Patil-Chhablani P, Venkatramani DV, Gandhi RA (2014). Ocular myasthenia gravis: a review. Indian J Ophthalmol.

[4] Koneczny I, Herbst R (2019). Myasthenia gravis: pathogenic effects of autoantibodies on neuromuscular architecture. Cells.

[5] Gilhus NE, Tzartos S, Evoli A, Palace J, Burns TM, Verschuuren JJ (2019). Myasthenia gravis. Nat Rev Dis Primers.

[6] Chaudhuri A, Behan PO (2009). Myasthenic crisis. QJM.

[7] Juel VC (2004). Myasthenia gravis: management of myasthenic crisis and perioperative care. Semin Neurol.

[8] Liu Y, Sawalha AH, Lu Q (2021). COVID-19 and autoimmune diseases. Curr Opin Rheumatol.

[9] Toussirot É, Bereau M (2015). Vaccination and induction of autoimmune diseases. Inflamm Allergy Drug Targets.

[10] Anzola AM, Trives L, Martínez-Barrio J, Pinilla B, Álvaro-Gracia JM, Molina-Collada J (2022). New-onset giant cell arteritis following COVID-19 mRNA (BioNTech/Pfizer) vaccine: a double-edged sword?. Clin Rheumatol.

[11] Tagliaferri AR, Narvaneni S, Azzam MH, Grist W (2021). A case of COVID-19 vaccine causing a myasthenia gravis crisis. Cureus.

[12] Ruan Z, Tang Y, Li C, Sun C, Zhu Y, Li Z, Chang T (2021). COVID-19 vaccination in patients with myasthenia gravis: a single-center case series. Vaccines (Basel).

[13] Moody R, Wilson K, Flanagan KL, Jaworowski A, Plebanski M (2021). Adaptive immunity and the risk of autoreactivity in COVID-19. Int J Mol Sci.

[14] Wraith DC, Goldman M, Lambert PH (2003). Vaccination and autoimmune disease: what is the evidence?. Lancet.

[15] Patone M, Mei XW, Handunnetthi L (2022). Risks of myocarditis, pericarditis, and cardiac arrhythmias associated with COVID-19 vaccination or SARS-CoV-2 infection. Nat Med.

[16] Jacob S, Muppidi S, Guidon A (2020). Guidance for the management of myasthenia gravis (MG) and Lambert-Eaton myasthenic syndrome (LEMS) during the COVID-19 pandemic. J Neurol Sci.

[17] Mehta P, McAuley DF, Brown M, Sanchez E, Tattersall RS, Manson JJ (2020). COVID-19: consider cytokine storm syndromes and immunosuppression. Lancet.

[18] Waggiallah HA (2022). Thrombosis formation after COVID-19 vaccination immunological aspects: review article. Saudi J Biol Sci.

[19] Stenton SB, Dalen D, Wilbur K (2005). Myocardial infarction associated with intravenous immune globulin. Ann Pharmacother.

[20] Shivamurthy P, Parker MW (2014). Cardiac manifestations of myasthenia gravis: a systematic review. IJC Metab Endocr.

